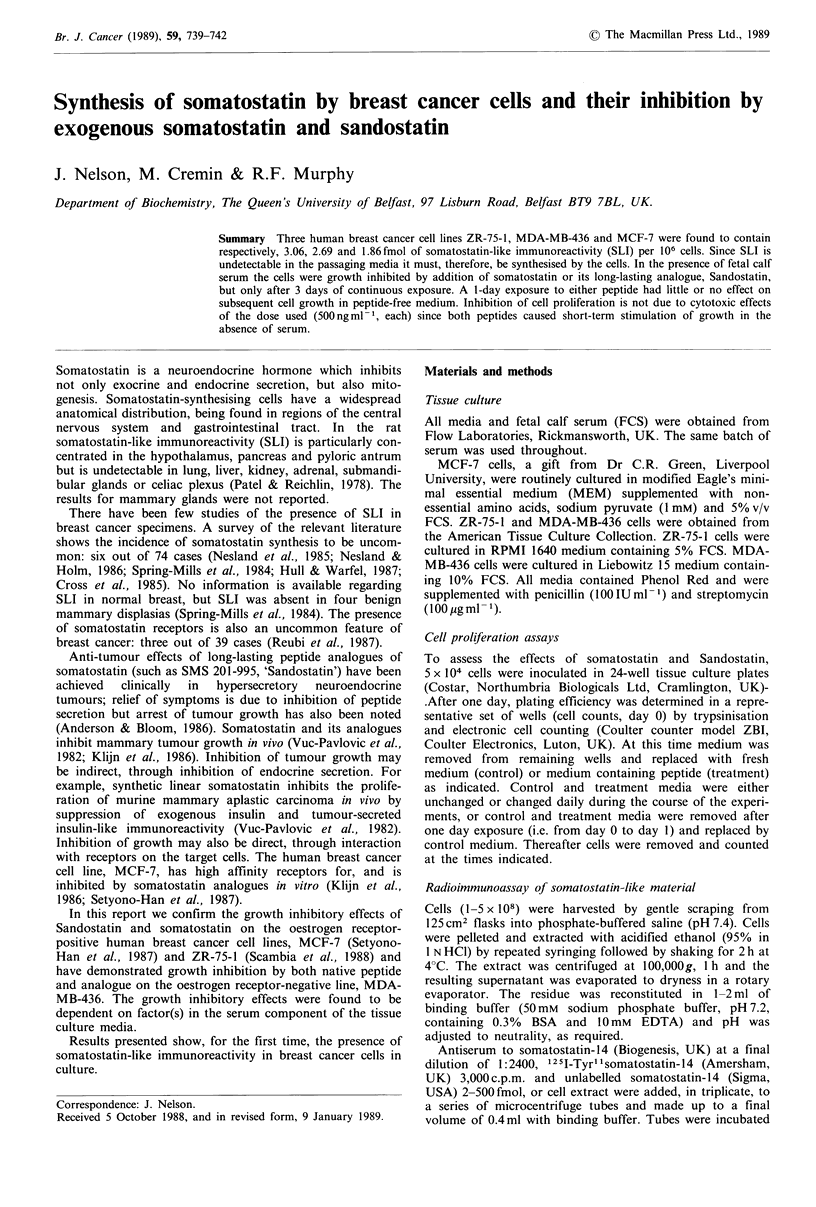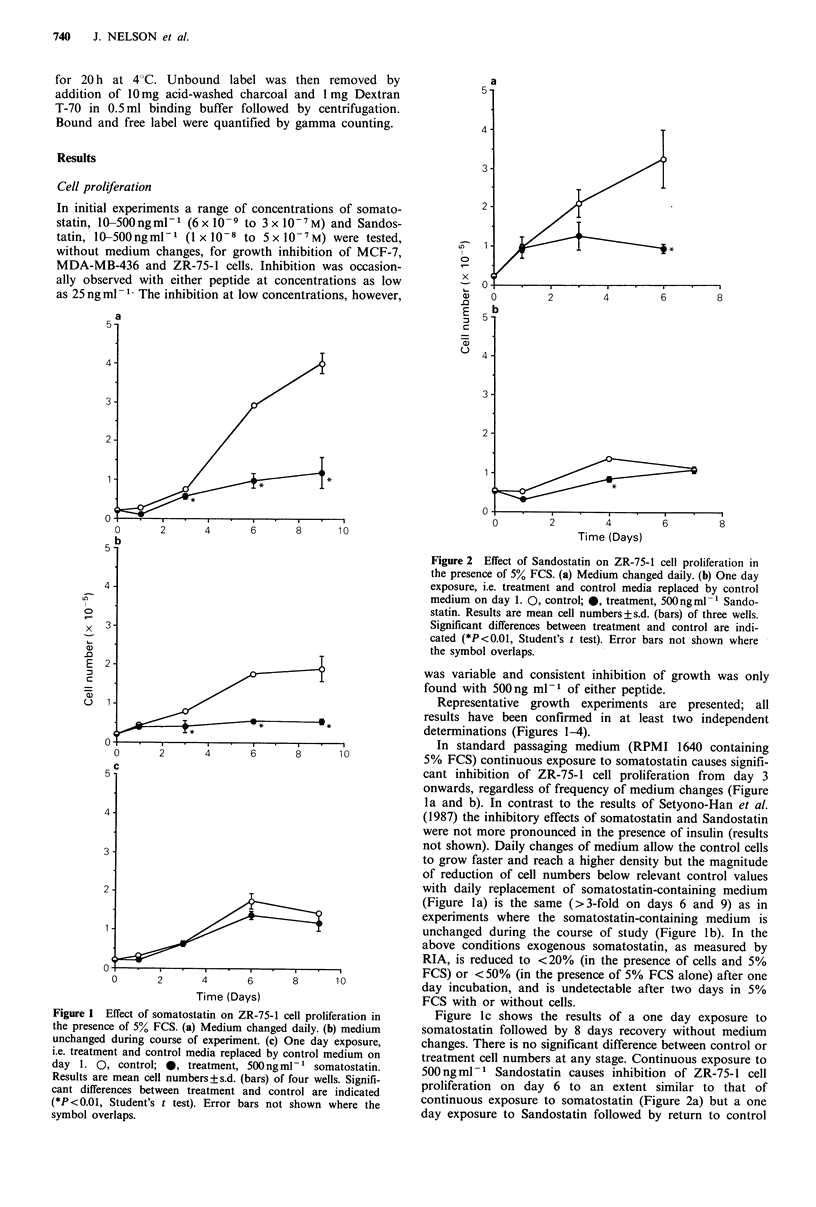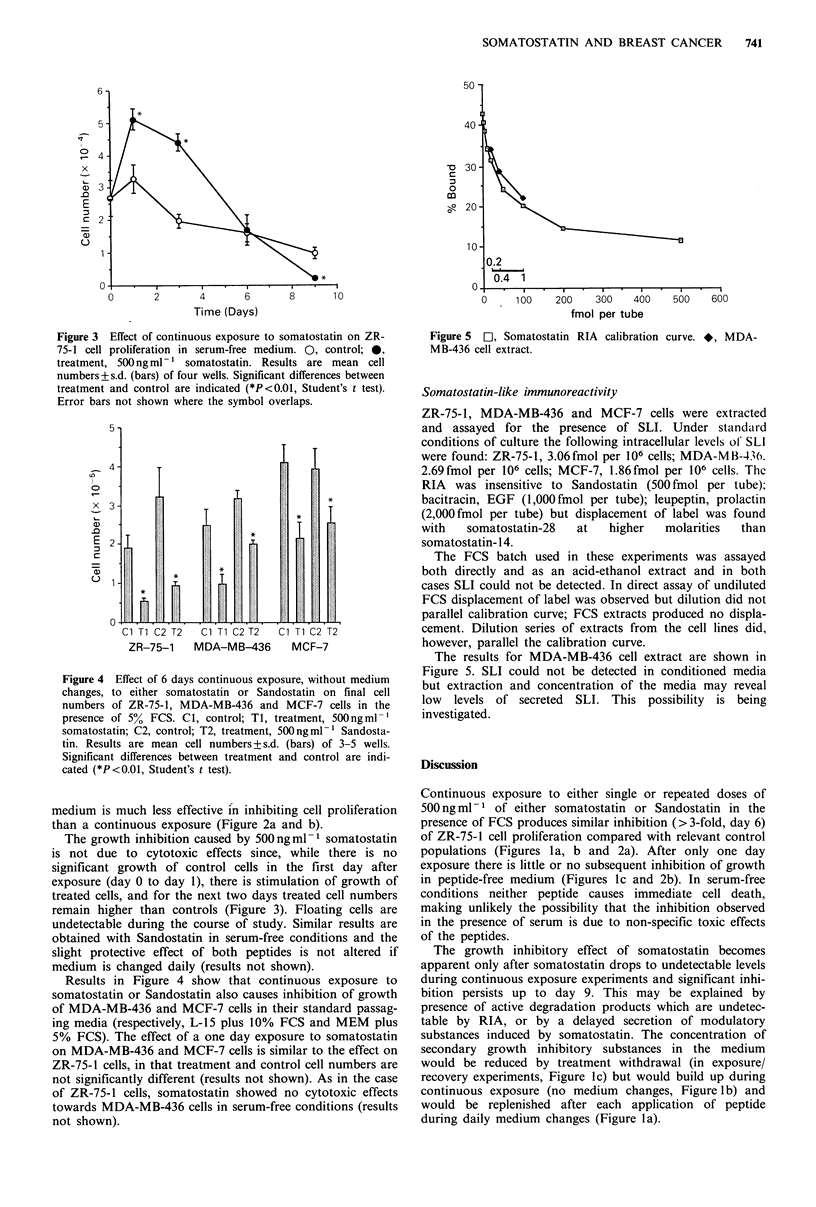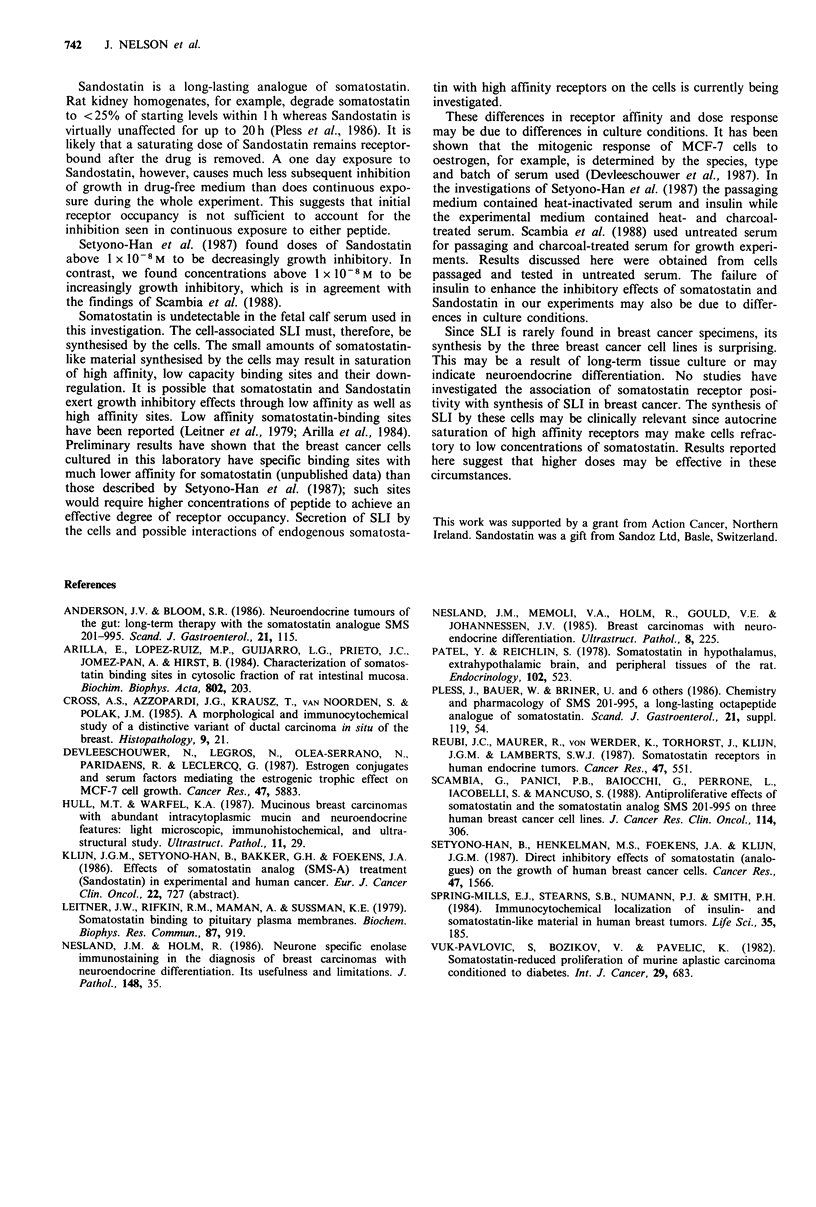# Synthesis of somatostatin by breast cancer cells and their inhibition by exogenous somatostatin and sandostatin.

**DOI:** 10.1038/bjc.1989.154

**Published:** 1989-05

**Authors:** J. Nelson, M. Cremin, R. F. Murphy

**Affiliations:** Department of Biochemistry, Queen's University of Belfast, UK.

## Abstract

Three human breast cancer cell lines ZR-75-1, MDA-MB-436 and MCF-7 were found to contain respectively, 3.06, 2.69 and 1.86 fmol of somatostatin-like immunoreactivity (SLI) per 10(6) cells. Since SLI is undetectable in the passaging media it must, therefore, be synthesised by the cells. In the presence of fetal calf serum the cells were growth inhibited by addition of somatostatin or its long-lasting analogue, Sandostatin, but only after 3 days of continuous exposure. A 1-day exposure to either peptide had little or no effect on subsequent cell growth in peptide-free medium. Inhibition of cell proliferation is not due to cytotoxic effects of the dose used (500 ng ml-1, each) since both peptides caused short-term stimulation of growth in the absence of serum.


Br. J. Cancer (1989), 59, 739–742 © The Macmillan Press Ltd., 1989

### Synthesis of somatostatin by breast cancer cells and their inhibition by exogenous somatostatin and sandostatin

J. Nelson, M. Cremin & R.F. Murphy

*Department of Biochemistry, The Queen's University of Belfast, 97 Lisburn Road, Belfast BT9 7BL, UK.*

**Summary** Three human breast cancer cell lines ZR-75-1, MDA-MB-436 and MCF-7 were found to contain respectively, 3.06, 2.69 and 1.86 fmol of somatostatin-like immunoreactivity (SLI) per $10^6$ cells. Since SLI is undetectable in the passaging media it must, therefore, be synthesised by the cells. In the presence of fetal calf serum the cells were growth inhibited by addition of somatostatin or its long-lasting analogue, Sandostatin, but only after 3 days of continuous exposure. A 1-day exposure to either peptide had little or no effect on subsequent cell growth in peptide-free medium. Inhibition of cell proliferation is not due to cytotoxic effects of the dose used (500 ng ml$^{-1}$, each) since both peptides caused short-term stimulation of growth in the absence of serum.

Somatostatin is a neuroendocrine hormone which inhibits not only exocrine and endocrine secretion, but also mitogenesis. Somatostatin-synthesising cells have a widespread anatomical distribution, being found in regions of the central nervous system and gastrointestinal tract. In the rat somatostatin-like immunoreactivity (SLI) is particularly concentrated in the hypothalamus, pancreas and pyloric antrum but is undetectable in lung, liver, kidney, adrenal, submandibular glands or celiac plexus (Patel & Reichlin, 1978). The results for mammary glands were not reported.

There have been few studies of the presence of SLI in breast cancer specimens. A survey of the relevant literature shows the incidence of somatostatin synthesis to be uncommon: six out of 74 cases (Nesland *et al.*, 1985; Nesland & Holm, 1986; Spring-Mills *et al.*, 1984; Hull & Warfel, 1987; Cross *et al.*, 1985). No information is available regarding SLI in normal breast, but SLI was absent in four benign mammary displasias (Spring-Mills *et al.*, 1984). The presence of somatostatin receptors is also an uncommon feature of breast cancer: three out of 39 cases (Reubi *et al.*, 1987).

Anti-tumour effects of long-lasting peptide analogues of somatostatin (such as SMS 201-995, 'Sandostatin') have been achieved clinically in hypersecretory neuroendocrine tumours; relief of symptoms is due to inhibition of peptide secretion but arrest of tumour growth has also been noted (Anderson & Bloom, 1986). Somatostatin and its analogues inhibit mammary tumour growth *in vivo* (Vuc-Pavlovic *et al.*, 1982; Klijn *et al.*, 1986). Inhibition of tumour growth may be indirect, through inhibition of endocrine secretion. For example, synthetic linear somatostatin inhibits the proliferation of murine mammary aplastic carcinoma *in vivo* by suppression of exogenous insulin and tumour-secreted insulin-like immunoreactivity (Vuc-Pavlovic *et al.*, 1982). Inhibition of growth may also be direct, through interaction with receptors on the target cells. The human breast cancer cell line, MCF-7, has high affinity receptors for, and is inhibited by somatostatin analogues *in vitro* (Klijn *et al.*, 1986; Setyono-Han *et al.*, 1987).

In this report we confirm the growth inhibitory effects of Sandostatin and somatostatin on the oestrogen receptor-positive human breast cancer cell lines, MCF-7 (Setyono-Han *et al.*, 1987) and ZR-75-1 (Scambia *et al.*, 1988) and have demonstrated growth inhibition by both native peptide and analogue on the oestrogen receptor-negative line, MDA-MB-436. The growth inhibitory effects were found to be dependent on factor(s) in the serum component of the tissue culture media.

Results presented show, for the first time, the presence of somatostatin-like immunoreactivity in breast cancer cells in culture.

Correspondence: J. Nelson.
Received 5 October 1988, and in revised form, 9 January 1989.

### Materials and methods

### *Tissue culture*

All media and fetal calf serum (FCS) were obtained from Flow Laboratories, Rickmansworth, UK. The same batch of serum was used throughout.

MCF-7 cells, a gift from Dr C.R. Green, Liverpool University, were routinely cultured in modified Eagle's minimal essential medium (MEM) supplemented with non-essential amino acids, sodium pyruvate (1 mM) and 5% v/v FCS. ZR-75-1 and MDA-MB-436 cells were obtained from the American Tissue Culture Collection. ZR-75-1 cells were cultured in RPMI 1640 medium containing 5% FCS. MDA-MB-436 cells were cultured in Liebowitz 15 medium containing 10% FCS. All media contained Phenol Red and were supplemented with penicillin (100 IU ml$^{-1}$) and streptomycin (100 $\mu$g ml$^{-1}$).

### *Cell proliferation assays*

To assess the effects of somatostatin and Sandostatin, $5 \times 10^4$ cells were inoculated in 24-well tissue culture plates (Costar, Northumbria Biologicals Ltd, Cramlington, UK)-.After one day, plating efficiency was determined in a representative set of wells (cell counts, day 0) by trypsinisation and electronic cell counting (Coulter counter model ZBI, Coulter Electronics, Luton, UK). At this time medium was removed from remaining wells and replaced with fresh medium (control) or medium containing peptide (treatment) as indicated. Control and treatment media were either unchanged or changed daily during the course of the experiments, or control and treatment media were removed after one day exposure (i.e. from day 0 to day 1) and replaced by control medium. Thereafter cells were removed and counted at the times indicated.

### *Radioimmunoassay of somatostatin-like material*

Cells ($1-5 \times 10^8$) were harvested by gentle scraping from 125 cm$^2$ flasks into phosphate-buffered saline (pH 7.4). Cells were pelleted and extracted with acidified ethanol (95% in 1 N HCl) by repeated syringing followed by shaking for 2 h at 4°C. The extract was centrifuged at 100,000 $g$, 1 h and the resulting supernatant was evaporated to dryness in a rotary evaporator. The residue was reconstituted in 1–2 ml of binding buffer (50 mM sodium phosphate buffer, pH 7.2, containing 0.3% BSA and 10 mM EDTA) and pH was adjusted to neutrality, as required.

Antiserum to somatostatin-14 (Biogenesis, UK) at a final dilution of 1:2400, $^{125}$I-Tyr$^{11}$somatostatin-14 (Amersham, UK) 3,000 c.p.m. and unlabelled somatostatin-14 (Sigma, USA) 2–500 fmol, or cell extract were added, in triplicate, to a series of microcentrifuge tubes and made up to a final volume of 0.4 ml with binding buffer. Tubes were incubated for 20 h at 4°C. Unbound label was then removed by addition of 10 mg acid-washed charcoal and 1 mg Dextran T-70 in 0.5 ml binding buffer followed by centrifugation. Bound and free label were quantified by gamma counting.

### Results

### *Cell proliferation*

In initial experiments a range of concentrations of somatostatin, 10–500 ng ml$^{-1}$ ($6 \times 10^{-9}$ to $3 \times 10^{-7}$ M) and Sandostatin, 10–500 ng ml$^{-1}$ ($1 \times 10^{-8}$ to $5 \times 10^{-7}$ M) were tested, without medium changes, for growth inhibition of MCF-7, MDA-MB-436 and ZR-75-1 cells. Inhibition was occasionally observed with either peptide at concentrations as low as 25 ng ml$^{-1}$. The inhibition at low concentrations, however,

**Figure 1** Effect of somatostatin on ZR-75-1 cell proliferation in the presence of 5% FCS. (**a**) Medium changed daily. (**b**) medium unchanged during course of experiment. (**c**) One day exposure, i.e. treatment and control media replaced by control medium on day 1. ○, control; ●, treatment, 500 ng ml$^{-1}$ somatostatin. Results are mean cell numbers ± s.d. (bars) of four wells. Significant differences between treatment and control are indicated (*$P<0.01$, Student's *t* test). Error bars not shown where the symbol overlaps.

**Figure 2** Effect of Sandostatin on ZR-75-1 cell proliferation in the presence of 5% FCS. (**a**) Medium changed daily. (**b**) One day exposure, i.e. treatment and control media replaced by control medium on day 1. ○, control; ●, treatment, 500 ng ml$^{-1}$ Sandostatin. Results are mean cell numbers ± s.d. (bars) of three wells. Significant differences between treatment and control are indicated (*$P<0.01$, Student's *t* test). Error bars not shown where the symbol overlaps.

was variable and consistent inhibition of growth was only found with 500 ng ml$^{-1}$ of either peptide.

Representative growth experiments are presented; all results have been confirmed in at least two independent determinations (Figures 1–4).

In standard passaging medium (RPMI 1640 containing 5% FCS) continuous exposure to somatostatin causes significant inhibition of ZR-75-1 cell proliferation from day 3 onwards, regardless of frequency of medium changes (Figure 1a and b). In contrast to the results of Setyono-Han *et al.* (1987) the inhibitory effects of somatostatin and Sandostatin were not more pronounced in the presence of insulin (results not shown). Daily changes of medium allow the control cells to grow faster and reach a higher density but the magnitude of reduction of cell numbers below relevant control values with daily replacement of somatostatin-containing medium (Figure 1a) is the same (>3-fold on days 6 and 9) as in experiments where the somatostatin-containing medium is unchanged during the course of study (Figure 1b). In the above conditions exogenous somatostatin, as measured by RIA, is reduced to <20% (in the presence of cells and 5% FCS) or <50% (in the presence of 5% FCS alone) after one day incubation, and is undetectable after two days in 5% FCS with or without cells.

Figure 1c shows the results of a one day exposure to somatostatin followed by 8 days recovery without medium changes. There is no significant difference between control or treatment cell numbers at any stage. Continuous exposure to 500 ng ml$^{-1}$ Sandostatin causes inhibition of ZR-75-1 cell proliferation on day 6 to an extent similar to that of continuous exposure to somatostatin (Figure 2a) but a one day exposure to Sandostatin followed by return to control

**Figure 3** Effect of continuous exposure to somatostatin on ZR-75-1 cell proliferation in serum-free medium. ○, control; ●, treatment, $500\,ng\,ml^{-1}$ somatostatin. Results are mean cell numbers ± s.d. (bars) of four wells. Significant differences between treatment and control are indicated (*$P<0.01$, Student's $t$ test). Error bars not shown where the symbol overlaps.

**Figure 4** Effect of 6 days continuous exposure, without medium changes, to either somatostatin or Sandostatin on final cell numbers of ZR-75-1, MDA-MB-436 and MCF-7 cells in the presence of 5% FCS. C1, control; T1, treatment, $500\,ng\,ml^{-1}$ somatostatin; C2, control; T2, treatment, $500\,ng\,ml^{-1}$ Sandostatin. Results are mean cell numbers ± s.d. (bars) of 3–5 wells. Significant differences between treatment and control are indicated (*$P<0.01$, Student's $t$ test).

medium is much less effective in inhibiting cell proliferation than a continuous exposure (Figure 2a and b).

The growth inhibition caused by $500\,ng\,ml^{-1}$ somatostatin is not due to cytotoxic effects since, while there is no significant growth of control cells in the first day after exposure (day 0 to day 1), there is stimulation of growth of treated cells, and for the next two days treated cell numbers remain higher than controls (Figure 3). Floating cells are undetectable during the course of study. Similar results are obtained with Sandostatin in serum-free conditions and the slight protective effect of both peptides is not altered if medium is changed daily (results not shown).

Results in Figure 4 show that continuous exposure to somatostatin or Sandostatin also causes inhibition of growth of MDA-MB-436 and MCF-7 cells in their standard passaging media (respectively, L-15 plus 10% FCS and MEM plus 5% FCS). The effect of a one day exposure to somatostatin on MDA-MB-436 and MCF-7 cells is similar to the effect on ZR-75-1 cells, in that treatment and control cell numbers are not significantly different (results not shown). As in the case of ZR-75-1 cells, somatostatin showed no cytotoxic effects towards MDA-MB-436 cells in serum-free conditions (results not shown).

**Figure 5** □, Somatostatin RIA calibration curve. ◆, MDA-MB-436 cell extract.

*Somatostatin-like immunoreactivity*

ZR-75-1, MDA-MB-436 and MCF-7 cells were extracted and assayed for the presence of SLI. Under standard conditions of culture the following intracellular levels of SLI were found: ZR-75-1, 3.06 fmol per $10^6$ cells; MDA-MB-436, 2.69 fmol per $10^6$ cells; MCF-7, 1.86 fmol per $10^6$ cells. The RIA was insensitive to Sandostatin (500 fmol per tube); bacitracin, EGF (1,000 fmol per tube); leupeptin, prolactin (2,000 fmol per tube) but displacement of label was found with somatostatin-28 at higher molarities than somatostatin-14.

The FCS batch used in these experiments was assayed both directly and as an acid-ethanol extract and in both cases SLI could not be detected. In direct assay of undiluted FCS displacement of label was observed but dilution did not parallel calibration curve; FCS extracts produced no displacement. Dilution series of extracts from the cell lines did, however, parallel the calibration curve.

The results for MDA-MB-436 cell extract are shown in Figure 5. SLI could not be detected in conditioned media but extraction and concentration of the media may reveal low levels of secreted SLI. This possibility is being investigated.

### Discussion

Continuous exposure to either single or repeated doses of $500\,ng\,ml^{-1}$ of either somatostatin or Sandostatin in the presence of FCS produces similar inhibition (>3-fold, day 6) of ZR-75-1 cell proliferation compared with relevant control populations (Figures 1a, b and 2a). After only one day exposure there is little or no subsequent inhibition of growth in peptide-free medium (Figures 1c and 2b). In serum-free conditions neither peptide causes immediate cell death, making unlikely the possibility that the inhibition observed in the presence of serum is due to non-specific toxic effects of the peptides.

The growth inhibitory effect of somatostatin becomes apparent only after somatostatin drops to undetectable levels during continuous exposure experiments and significant inhibition persists up to day 9. This may be explained by presence of active degradation products which are undetectable by RIA, or by a delayed secretion of modulatory substances induced by somatostatin. The concentration of secondary growth inhibitory substances in the medium would be reduced by treatment withdrawal (in exposure/recovery experiments, Figure 1c) but would build up during continuous exposure (no medium changes, Figure 1b) and would be replenished after each application of peptide during daily medium changes (Figure 1a).

Sandostatin is a long-lasting analogue of somatostatin. Rat kidney homogenates, for example, degrade somatostatin to <25% of starting levels within 1 h whereas Sandostatin is virtually unaffected for up to 20 h (Pless *et al.*, 1986). It is likely that a saturating dose of Sandostatin remains receptor-bound after the drug is removed. A one day exposure to Sandostatin, however, causes much less subsequent inhibition of growth in drug-free medium than does continuous exposure during the whole experiment. This suggests that initial receptor occupancy is not sufficient to account for the inhibition seen in continuous exposure to either peptide.

Setyono-Han *et al.* (1987) found doses of Sandostatin above $1 \times 10^{-8}$ M to be decreasingly growth inhibitory. In contrast, we found concentrations above $1 \times 10^{-8}$ M to be increasingly growth inhibitory, which is in agreement with the findings of Scambia *et al.* (1988).

Somatostatin is undetectable in the fetal calf serum used in this investigation. The cell-associated SLI must, therefore, be synthesised by the cells. The small amounts of somatostatin-like material synthesised by the cells may result in saturation of high affinity, low capacity binding sites and their down-regulation. It is possible that somatostatin and Sandostatin exert growth inhibitory effects through low affinity as well as high affinity sites. Low affinity somatostatin-binding sites have been reported (Leitner *et al.*, 1979; Arilla *et al.*, 1984). Preliminary results have shown that the breast cancer cells cultured in this laboratory have specific binding sites with much lower affinity for somatostatin (unpublished data) than those described by Setyono-Han *et al.* (1987); such sites would require higher concentrations of peptide to achieve an effective degree of receptor occupancy. Secretion of SLI by the cells and possible interactions of endogenous somatostatin with high affinity receptors on the cells is currently being investigated.

These differences in receptor affinity and dose response may be due to differences in culture conditions. It has been shown that the mitogenic response of MCF-7 cells to oestrogen, for example, is determined by the species, type and batch of serum used (Devleeschouwer *et al.*, 1987). In the investigations of Setyono-Han *et al.* (1987) the passaging medium contained heat-inactivated serum and insulin while the experimental medium contained heat- and charcoal-treated serum. Scambia *et al.* (1988) used untreated serum for passaging and charcoal-treated serum for growth experiments. Results discussed here were obtained from cells passaged and tested in untreated serum. The failure of insulin to enhance the inhibitory effects of somatostatin and Sandostatin in our experiments may also be due to differences in culture conditions.

Since SLI is rarely found in breast cancer specimens, its synthesis by the three breast cancer cell lines is surprising. This may be a result of long-term tissue culture or may indicate neuroendocrine differentiation. No studies have investigated the association of somatostatin receptor positivity with synthesis of SLI in breast cancer. The synthesis of SLI by these cells may be clinically relevant since autocrine saturation of high affinity receptors may make cells refractory to low concentrations of somatostatin. Results reported here suggest that higher doses may be effective in these circumstances.

This work was supported by a grant from Action Cancer, Northern Ireland. Sandostatin was a gift from Sandoz Ltd, Basle, Switzerland.

### References

ANDERSON, J.V. & BLOOM, S.R. (1986). Neuroendocrine tumours of the gut: long-term therapy with the somatostatin analogue SMS 201-995. *Scand. J. Gastroenterol.*, **21**, 115.

ARILLA, E., LOPEZ-RUIZ, M.P., GUIJARRO, L.G., PRIETO, J.C., JOMEZ-PAN, A. & HIRST, B. (1984). Characterization of somatostatin binding sites in cytosolic fraction of rat intestinal mucosa. *Biochim. Biophys. Acta*, **802**, 203.

CROSS, A.S., AZZOPARDI, J.G., KRAUSZ, T., VAN NOORDEN, S. & POLAK, J.M. (1985). A morphological and immunocytochemical study of a distinctive variant of ductal carcinoma *in situ* of the breast. *Histopathology*, **9**, 21.

DEVLEESCHOUWER, N., LEGROS, N., OLEA-SERRANO, N., PARIDAENS, R. & LECLERCQ, G. (1987). Estrogen conjugates and serum factors mediating the estrogenic trophic effect on MCF-7 cell growth. *Cancer Res.*, **47**, 5883.

HULL, M.T. & WARFEL, K.A. (1987). Mucinous breast carcinomas with abundant intracytoplasmic mucin and neuroendocrine features: light microscopic, immunohistochemical, and ultrastructural study. *Ultrastruct. Pathol.*, **11**, 29.

KLIJN, J.G.M., SETYONO-HAN, B., BAKKER, G.H. & FOEKENS, J.A. (1986). Effects of somatostatin analog (SMS-A) treatment (Sandostatin) in experimental and human cancer. *Eur. J. Cancer Clin. Oncol.*, **22**, 727 (abstract).

LEITNER, J.W., RIFKIN, R.M., MAMAN, A. & SUSSMAN, K.E. (1979). Somatostatin binding to pituitary plasma membranes. *Biochem. Biophys. Res. Commun.*, **87**, 919.

NESLAND, J.M. & HOLM, R. (1986). Neurone specific enolase immunostaining in the diagnosis of breast carcinomas with neuroendocrine differentiation. Its usefulness and limitations. *J. Pathol.*, **148**, 35.

NESLAND, J.M., MEMOLI, V.A., HOLM, R., GOULD, V.E. & JOHANNESSEN, J.V. (1985). Breast carcinomas with neuroendocrine differentiation. *Ultrastruct. Pathol.*, **8**, 225.

PATEL, Y. & REICHLIN, S. (1978). Somatostatin in hypothalamus, extrahypothalamic brain, and peripheral tissues of the rat. *Endocrinology*, **102**, 523.

PLESS, J., BAUER, W. & BRINER, U. and 6 others (1986). Chemistry and pharmacology of SMS 201-995, a long-lasting octapeptide analogue of somatostatin. *Scand. J. Gastroenterol.*, **21**, suppl. 119, 54.

REUBI, J.C., MAURER, R., VON WERDER, K., TORHORST, J., KLIJN, J.G.M. & LAMBERTS, S.W.J. (1987). Somatostatin receptors in human endocrine tumors. *Cancer Res.*, **47**, 551.

SCAMBIA, G., PANICI, P.B., BAIOCCHI, G., PERRONE, L., IACOBELLI, S. & MANCUSO, S. (1988). Antiproliferative effects of somatostatin and the somatostatin analog SMS 201-995 on three human breast cancer cell lines. *J. Cancer Res. Clin. Oncol.*, **114**, 306.

SETYONO-HAN, B., HENKELMAN, M.S., FOEKENS, J.A. & KLIJN, J.G.M. (1987). Direct inhibitory effects of somatostatin (analogues) on the growth of human breast cancer cells. *Cancer Res.*, **47**, 1566.

SPRING-MILLS, E.J., STEARNS, S.B., NUMANN, P.J. & SMITH, P.H. (1984). Immunocytochemical localization of insulin- and somatostatin-like material in human breast tumors. *Life Sci.*, **35**, 185.

VUK-PAVLOVIC, S, BOZIKOV, V. & PAVELIC, K. (1982). Somatostatin-reduced proliferation of murine aplastic carcinoma conditioned to diabetes. *Int. J. Cancer*, **29**, 683.